# Central role of myeloid MCPIP1 in protecting against LPS-induced inflammation and lung injury

**DOI:** 10.1038/sigtrans.2017.66

**Published:** 2017-12-08

**Authors:** Yong Li, Xuan Huang, Shengping Huang, Hui He, Tianhua Lei, Fatma Saaoud, Xiao-Qiang Yu, Ari Melnick, Anil Kumar, Christopher J Papasian, Daping Fan, Mingui Fu

**Affiliations:** 1 Department of Biomedical Science and Shock/Trauma Research Center, School of Medicine, University of Missouri-Kansas City, Kansas City, Missouri, MO, USA; 2 Institute of Translational Medicine, Nanchang University, Nanchang, Jiangxi, P. R. China; 3 Department of Cell Biology and Anatomy, University of South Carolina School of Medicine, Columbia, SC, USA; 4 Division of Molecular Biology and Biochemistry, School of Biological Sciences, University of Missouri-Kansas City, Kansas City, Missouri, MO, USA; 5 Division of Hematology and Medical Oncology, Weill Cornell Medical College, New York, NY, USA; 6 Division of Pharmacology and Toxicology, School of Pharmacy, University of Missouri-Kansas City, Kansas City, Missouri, MO, USA

## Abstract

Although systemic inflammatory responses attributable to infection may lead to significant lung injury, the precise molecular mechanisms leading to lung damage are poorly understood and therapeutic options remain limited. Here, we show that myeloid monocyte chemotactic protein-inducible protein 1 (MCPIP1) plays a central role in protecting against LPS-induced inflammation and lung injury. Myeloid-specific MCPIP1 knockout mice developed spontaneous inflammatory syndromes, but at a late age compared to global MCPIP1 knockout mice. Moreover, mice with a myeloid-specific deletion of MCPIP1 were extremely sensitive to LPS-induced lung injury due to overproduction of proinflammatory cytokines and chemokines. We identified C/EBPβ and C/EBPδ, two critical transcriptional factors that drive cytokine production and lung injury, as targets of MCPIP1 RNase. LPS administration caused MCPIP1 protein degradation in the lungs. Pharmacological inhibition of MALT1, a paracaspase that cleaves MCPIP1, by MI-2 selectively increased the MCPIP1 protein levels in macrophages and in the lungs. Meanwhile, administration of MI-2 protected mice from LPS-induced inflammation, lung injury and death. Collectively, these results indicate that myeloid MCPIP1 is central in controlling LPS-induced inflammation and lung injury. Pharmacological inhibition of MALT1 protease activity may be a good strategy to treat inflammatory diseases by enhancing MCPIP1 expression in myeloid cells.

## Introduction

The primary benefit derived from the host inflammatory response to infection is the potential eradication or containment of invading microbes. Significant cell damage and destruction are other consequences of the inflammatory response to infection, and when these inflammatory responses become systemic (that is, sepsis), multi-organ failure and death are common outcomes.^1^ Acute lung injury, a syndrome characterized by pulmonary inflammation and vascular hyperpermeability, is a particularly common finding in sepsis, substantially contributing to mortality.^2,3^ The basic pathological mechanism of sepsis-induced lung injury involves the destruction of pulmonary capillaries and alveolar epithelia.^4–8^

The host inflammatory response is mediated by cytokines, and their production is precisely regulated to permit rapid, robust responses to invading microbes, along with attenuation of those responses once the threat has been contained.^9,10^ Lipopolysaccharide (LPS) is a component of the outer membrane of Gram-negative bacteria; it is among the most potent triggers of host inflammatory responses, and LPS-induced cytokine production is regulated at both the transcriptional and post-transcriptional levels. Transcription of cytokine genes is controlled by the following three transcription factors: NF-κB, C/EBPδ and ATF-3. The activation of NF-κB triggers early induction of LPS-responsive genes. C/EBPδ further enhances the gene transcription and acts together with NF-κB to obtain maximal transcription of those genes, thereby contributing to the amplification and persistence of inflammation. Meanwhile, NF-κB induces transcription of ATF-3. ATF-3 subsequently suppresses C/EBPδ transcription, by which it suppresses the inflammatory response.^11,12^

Although transcriptional regulation of inflammatory gene expression has been extensively studied, post-transcriptional regulation remains largely unknown. MCPIP1, also known as regnase-1 and Zc3h12a, was initially identified as the most highly induced protein by monocyte chemotactic protein 1 (MCP1).^13^ MCPIP1 is an endonuclease that selectively promotes destabilization of mRNAs that encode certain inflammatory cytokines, signal transducers and transcription factors.^14–18^ Through this central mechanism, MCPIP1 serves as an essential regulator of inflammatory cell activation and immune homeostasis.^19–21^

The biological significance of MCPIP1 has been demonstrated in MCPIP1 global knockout mice. These mice develop abnormal responses in both innate and adaptive immune cells, as manifested by splenomegaly, lymphadenopathy, heightened cytokine production and multi-organ inflammation, particularly in the lungs.^22–24^ The specific roles of myeloid MCPIP1 in the pathogenesis of inflammatory diseases, however, remain unknown. The goal of the current study was to determine the specific role of myeloid MCPIP1 in LPS-induced inflammation, with an emphasis on the pathological consequences in the lungs. To accomplish this, we generated myeloid-specific MCPIP1 knockout mice (M-Mcpip1^−/−^) by crossing a floxed mouse line with mice expressing the Cre recombinase transgene under the control of the Lysozyme M promoter. We also characterized the therapeutic potential of enhancing the MCPIP1 protein levels in protecting against LPS-induced lung injury.

## Methods

### Generation of mice with a Floxed Mcpip1 allele and cell-specific knockouts

**Figure 1:**
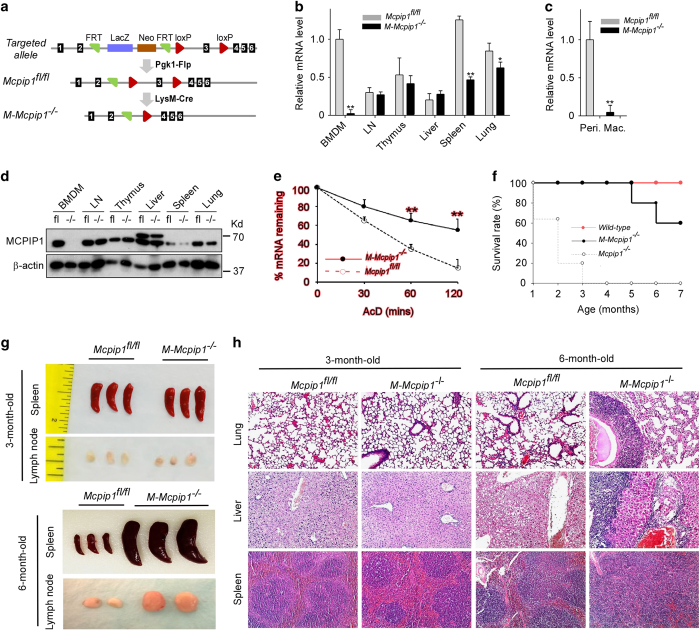
Generation and characterization of M-Mcpip1^−/−^ mice. (**a**) Schematic map of the strategy used for generating the Mcpip1 conditional targeted allele, Mcpip1^floxflox^, and M-Mcpip1^−/−^ mice. (**b**) qPCR analysis of the MCPIP1 mRNA levels in BMDMs and the indicated tissues collected from Mcpip1^flfl^ and M-Mcpip1^−/−^ mice. Data are representative of three independent experiments with 3 mice per group. *P<0.05, **P<0.001 by Student’s *t*-test. (**c**) qPCR analysis of the MCPIP1 mRNA levels in peritoneal macrophages collected from Mcpip1^flfl^ and M-Mcpip1^−/−^ mice. Data are representative of three independent experiments with 3 mice per group. **P<0.001 by Student’s *t*-test. (**d**) Immunoblot analysis of the MCPIP1 protein levels in BMDMs and indicated tissues from Mcpip1^flfl^ and M-Mcpip1^−/−^ mice. β-actin served as a loading control. Data are representative of three independent experiments. (**e**) BMDMs from Mcpip1^flfl^ and M-Mcpip1^−/−^ mice were stimulated with LPS (1 μg/ml) for 4 h and then incubated with AcD (5 μg/ml) for different time points as indicated. The mRNA levels of IL-6 were measured by qPCR and normalized as 100% at the 0-hr time point. Data were presented as the means±s.d., *n*=4. (**f**) Survival rate of Mcpip1 global knockout (Mcpip1^−/−^) mice, myeloid-specific Mcpip1 knockout (M-Mcpip1^−/−^) mice and wild-type mice (*n*=10). (**g**) Photographs of spleens and lymph nodes collected from Mcpip1^flfl^ and M-Mcpip1^−/−^ mice at 3 months and 6 months of age. (**h**) Hematoxylin-and-eosin staining of lung and liver sections from Mcpip1^flfl^ and M-Mcpip1^−/−^ mice at 4 months of age or 6 months of age, respectively. Original magnification is 100×. Data are representative of three independent experiments.

Two C57BL/6 mouse embryonic stem cell clones (HEPD0517-2-B05 and HEPD0517-2-G04) containing MCPIP1 conditional gene knock-out mutations were purchased from the European Conditional Mouse Mutagenesis Program (EUCOMM) and confirmed by Southern blot. These clones were sent to the University of Missouri Transgenic Animal Core, which produced heterozygous mice with the targeted allele. Then, we crossed this mouse line with a Pgk-Flp transgenic mouse line to remove the lacZ-neo cassette from the mouse genome and obtained a Mcpip1^flox/+^ mouse line, with two loxp sites flanking the two sides of exon 3 of the Mcpip1 gene (Figure 1a). This mouse line was further interbred to produce Mcpip1^fl/fl^ mice. To produce myeloid-specific MCPIP1 knockout mice, MCPIP1^fl/fl^ mice were crossed with myeloid-specific *Cre-*expressing transgenic mice (LysM-Cre, Stock#: 004781, Jackson Laboratory). The resultant mice were designated as M-Mcpip1^−/−^. Genotyping for loxP insertion was performed by PCR using the following primers: GCCTTCCTGATCCTATTGGAG (wild-type), GAGATGGCGCAGCGCAATTAAT (knock-out) and GCCTCTTGTCACTCCCTCCTCC (common). Genotyping for LysMCre included the following primers: TTACAGTCGGCCAGGCTGAC (wild-type), CCCAGAAATGCCAGATTACG (mutant) and CTTGGGCTGCCAGAATTTCTC (common). The mice were housed in a pathogen-free animal facility at 25 °C and were illuminated by 12:12-h light-dark cycles. The mice were provided with standard rodent chow and water *ad libitum*. All of the animal breeding and other procedures were approved by the Institutional Animal Care and Use Committee of University of Missouri Kansas City and followed the Institutional and US National Institutes of Health guidelines.

### LPS challenge in mice

Mcpip1^fl/fl^ and M-Mcpip1^−/−^ mice at 2-months of age were used. Mice were challenged by i.p. injection of LPS at a dose of 25 mg/kg (O127:B8; Sigma-Aldrich) in 200 μl sterile saline. After injection, the mice were closely monitored for general condition and survival. At 8 h after LPS challenge, the mice were euthanized, and serum and organs were collected for ELISA analyses and histological examinations. For the study of the effects of MI-2 on mice survival, C57BL/6 mice from the Jackson Laboratory were divided into two groups: one group was only injected with a lethal dose of LPS (40 mg/kg body weight, i.p.); the other group was pre-treated with MI-2 (25 mg/Kg body weight, i.p.) at 2 h before LPS injection and another dose at 2 h post-LPS injection. All mice were closely monitored, and a ‘moribund status’ was equated with death to minimize the discomfort of the mice. The survival rate was recorded. For the study of MI-2 on LPS-induced inflammation and lung injury, adult C57BL/6 mice were divided into four groups. The first group of mice were treated with MI-2 (25 mg/body weight, i.p.) for 2 h, then the mice were treated with LPS (25 mg/kg body weight, i.p.); the second group of mice were treated with PBS for 2 h, then the mice were treated with LPS; the third group of mice was injected with LPS for 2 h and then treated with MI-2; the fourth group of mice were treated with PBS only. All mice were sacrificed after 8 h of LPS injection, and the sera and lungs were collected for ELISA, immunoblot and qPCR analysis. All experiments were approved by the Institutional Animal Care and Use Committee of University of Missouri Kansas City.

### Cells

The mouse macrophage RAW264.7 cell line and the human THP1 monocyte cell line were obtained from the American Type Culture Collection and maintained in RPMI 1640 medium supplemented with 2 mM Glutamine, 100 U/ml penicillin and streptomycin, and 10% FBS (endotoxin <1 ng/ml; Sigma-Aldrich). Mouse BMDMs were generated from BM stem cells that were obtained from femurs of male Mcpip1^fl/fl^ and M-Mcpip1^−/−^ mice, as described previously.^23^ Briefly, mice were euthanized by CO_2_ inhalation, and bone marrow cells were isolated from femurs. After overnight culture, nonadherent bone marrow cells were collected and cultured in RPMI 1640 medium supplemented with 10% heat-inactivated FBS, 25 mM HEPES, 2 mM Glutamine, 100 U/ml penicillin, 100 μg/ml streptomycin, plus 30% (v/v) L929 cell-conditioned medium. Adherent macrophage monolayers were obtained and their purity were determined by flow cytometric analysis. Differentiated BMDMs were then harvested by gently scraping the cells from the dishes using a rubber spatula and seeded onto 100-mm petri dishes for experiments. Cells were subjected to serum starvation for 16 h in macrophage serum-free medium and then treated with 1 μg/ml LPS for different time points. Human primary monocytes were purchased from Lonza and cultured with the specific medium provided by Lonza. The primary peritoneal macrophages were isolated by flushing the peritoneal cavity with PBS.

### Reagents

pCMV-luc-C/EBPδ3′UTR were purchased from GeneCopoeia. A plasmid containing human C/EBPδ cDNA and full-length 3′UTR was kindly provided by Dr Esta Sterneck (NCI/NIH). Using this plasmid as a template, PCR was performed to amplify the fragments of the human C/EBPδ 3′UTR, and these were inserted into pCMV-luc-control vector by EcoR I/Xho I. MCPIP1 rabbit polyclonal antibody was from GeneTex (GTX110807). C/EBPβ, C/EBPδ, C/EBPα, MALT1, BCL10, A20, CYLD, RelB and β-actin antibodies were purchased from Cell Signaling Technology. LPS (L3129, O127:B8) was purchased from Sigma. SiRNAs targeting MCPIP1 and MALT1 were from Santa Cruz Biotechnology. qPCR primers were ordered from IDT. MI-2, anti-F4/80 and anti-Gr1 antibodies were purchased from R&D Systems.

### Histological and immunohistochemical analysis

Tissues were fixed in 4% phosphate-buffered paraformaldehyde and processed using standard procedures. 5-μm paraffin tissue sections were collected and stained with H&E. For immunostaining, sections were incubated in 3% H_2_O_2_ to inactivate endogenous peroxidases, followed by Ag retrieval with heat and pressure in citrate buffer. Endogenous biotin was blocked with blocking reagents. Sections were then incubated with anti-F4/80 and anti-Gr1 Abs for 60 min at a 1:250 dilution, followed by HRP-conjugated secondary antibodies for 30 min. Immunolabeled Ag-Ab complexes were visualized using SignalStain DAB substrate kit (Cell Signaling Tech.). The sections were lightly counterstained with hematoxylin before analysis.

### Transient transfection and luciferase assay

Transfection and luciferase assay were performed as described previously.^13^ All transfections were performed in triplicate and repeated at least two times.

### Protein isolation and immunoblotting

Protein isolation and immunoblotting were essentially performed as described previously.^13^ Tissue extracts and whole-cell lysates were prepared in RIPA buffer (Sigma) containing protease inhibitors (Complete Mini; Roche, Indianapolis, IN), respectively, cleared by centrifugation, and quantified using the Bradford assay. Denatured lysates were separated on 8–12% Tris-HCl gels and transferred onto nitrocellulose membranes. Blots were incubated at 4 °C overnight with antibodies as indicated, followed by incubation with HRP-conjugated goat anti-rabbit IgG. Immune complexes were detected using ECL chemiluminescent substrate.

### Measurement of cytokine levels in sera

Mouse serum levels of cytokines/chemokines were measured using a Bio-Plex Pro^TM^ Mouse Cytokine 23-plex Assay Kit (Bio-Rad) on a Bio-Plex system following the manufacturer’s instructions.

### RNA isolation and quantitative PCR (qPCR)

Total tissue or cellular RNA was isolated using TRIzol reagent, according to the manufacturer’s instructions (Invitrogen). Residual genomic DNA was removed by on-column digestion with RNase-free DNase I. To determine the decay rates of cytokine transcripts, actinomycin D (5 μg/ml; Sigma-Aldrich) was added to block transcription after BMDMs were stimulated with LPS (1 μg/ml) for 4 h. Total RNA was isolated at the indicated times over a 2-h time course after the addition of actinomycin D. IL-6, C/EBPβ and C/EBPδ transcript levels were determined by qPCR. First-strand cDNA was synthesized using oligo(dT)_12-18_ primers and SuperScript III Reverse Transcriptase. Real-time PCR was performed using SYBR Green and the ABI Step-One Plus Detection System (Applied Biosystems). Primers were designed and compared with the current mouse genome reference sequence using BLAST to ensure that no cross-reactivity with other genes would occur. The results were normalized against the β-actin transcript as an internal control and were then used to calculate expression levels according to the ΔΔ cycle threshold method. All data were expressed in terms of fold-change relative to the unstimulated sample, which was set as 1, unless otherwise specified. The primers were validated for their amplification efficiency and specificity prior to being used in the study. The primer sequences are presented in Supplementary Table 1.

### Statistics

Data were expressed as the means±s.d. For comparison between two groups, the unpaired Student’s *t* test was used. For multiple comparisons, analysis of variance followed by unpaired Student’s *t* test was used. For mouse survival rates, the log-rank Mantel-Cox test was used. A value of *P*<0.05 was considered statistically significant.

## Results

### Myeloid-specific MCPIP1 knockout mice develop late-onset inflammatory syndrome

To study cell-specific functions of MCPIP1 *in vivo*, we generated a Mcpip1-floxed mouse line in a C57BL/6 background. As shown in Figure 1a, the targeted allele contains two loxp sites flanking the two sides of exon 3 of the Mcpip1 gene. After crossing with a Pgk-Flp transgenic mouse line to remove the lacZ-neo cassette, we obtained a Mcpip1^fl/+^ mouse line. This mouse line was further interbred to produce Mcpip1^fl/fl^ mice. By crossing Mcpip1^fl/fl^ mice with myeloid-specific transgenic mice of *Cre* (LysM-Cre, Stock#: 004781, Jackson Labs),^25^ we generated myeloid-specific MCPIP1 knockout (M-Mcpip1^−/−^) mice. To verify that the Mcpip1 gene was successfully and specifically deleted in myeloid cells, such as macrophages, we isolated bone-marrow derived macrophages (BMDMs) and other tissues from both Mcpip1^fl/fl^ and M-Mcpip1^−/−^ mice. Real-time quantitative PCR (qPCR) analysis showed specific deletion of MCPIP1 mRNA in BMDMs, but not in thymus and lymph node tissues, which predominantly contain lymphocytes (Figure 1b). Deletion of MCPIP1 mRNA was further confirmed in primary peritoneal macrophages by qPCR (Figure 1c). Immunoblot analysis further showed that the MCPIP1 protein was specifically deleted in BMDMs, but not in liver, thymus and lymph node tissues (Figure 1d). Both the MCPIP1 mRNA and protein levels were also decreased to an extent in the spleen and lungs, likely attributable to the high numbers of macrophages present in these tissues (Figures 1b and d). IL-6 mRNA is a known target of MCPIP1 RNase.^22^ As shown in Figure 1e, the decay of IL-6 mRNA was significantly delayed in M-Mcpip1^−/−^ BMDMs, suggesting that the MCPIP1 is functionally lost in knockout macrophages. These results confirmed that the Mcpip1 gene was specifically deleted in macrophages from M-Mcpip1^−/−^ mice.

M-Mcpip1^−/−^ mice were born at the expected Mendelian ratio and had no gross abnormalities up to 5 months of age. However, after 5 months of age, mice failed to thrive and developed cachexia (decreased body weight), splenomegaly, lymphadenopathy, multi-organ inflammation and premature death, comparable to that observed in MCPIP1 global knockout mice, but at a much older age. As shown in Figure 1f, global MCPIP1 knockout mice all died within 4 months, whereas myeloid-specific MCPIP1 knockout mice began to die after 5 months, and 60% of these mice were alive at 7 months of age. At the same time, all wild-type mice survived. The sizes of the spleens and lymph nodes of 3-month-old M-Mcpip1^−/−^ mice appeared normal, but M-Mcpip1^−/−^ mice developed spontaneous splenomegaly and lymphadenopathy at 6 months of age (Figure 1g). As shown in Figure 1h, minimal inflammation was observed in the lungs and liver of M-Mcpip1^−/−^ mice at 3 months of age, whereas inflammatory cell infiltration was marked in the lungs and liver from M-Mcpip1^−/−^ mice at 6 months of age. Similarly, minimal architectural changes in the spleens of M-Mcpip1^−/−^ mice were observed at 3 months of age, whereas the architecture of the spleen of M-Mcpip1^−/−^ mice at 6 months of age was disorganized due to marked expansion of the white pulp. A more detailed phenotypic characterization of M-Mcpip1^−/−^ mice is being performed and will be reported elsewhere.

### Myeloid MCPIP1 suppresses LPS-induced inflammation and lung injury

**Figure 2:**
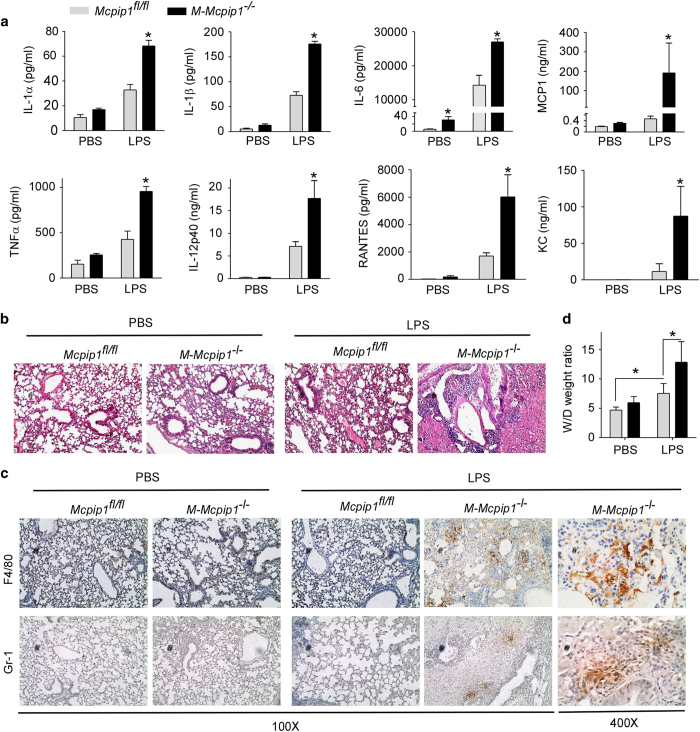
Mice with myeloid-specific knockout of MCPIP1 are susceptible to LPS-induced inflammation and lung injury. (**a**) Serum levels of proinflammatory cytokines and chemokines in Mcpip1^flfl^ and M-Mcpip1^−/−^ mic, treated intraperitoneally with PBS or LPS for 8 h. Data are presented as the means±s.d. (*n*=5). *P<0.05, versus Mcpip1^flfl^ group. (**b**) H&E staining of lung sections from Mcpip1^flfl^ and M-Mcpip1^−/−^ mice at 2 months of age treated intraperitoneally with PBS or LPS for 8 h. Original magnification is 100×. Data are representative of three independent experiments. (**c**) Immunohistological staining of lung sections from Mcpip1^flfl^ and M-Mcpip1^−/−^ mice at 2 months of age treated intraperitoneally with PBS or LPS for 8 h, with anti-F4/80 or Gr-1 antibodies. Original magnification is 100× or 400×, as indicated. Data are representative of three independent experiments. (**d**) Changes of lung wet-to-dry ratios in Mcpip1^flfl^ and M-Mcpip1^−/−^ mice treated intraperitoneally with PBS or LPS for 8 h. Data are presented as the means±s.d., *P<0.05 by Student’s *t*-test.

To determine whether expression of MCPIP1 in myeloid cells is critical for protecting against LPS-induced inflammation and lung injury, 2-month-old Mcpip1^fl/fl^ and M-Mcpip1^−/−^ mice were injected with LPS (25 mg/kg body weight, i.p.). After 8 h, mice were euthanized, serum was collected to measure cytokines, and the lungs were fixed and analyzed by H&E staining. As shown in Figure 2a, LPS inoculation induced higher serum concentrations of proinflammatory cytokines and chemokines, including IL-1α, IL-1β, IL-6, IL-12p40, TNFα, MCP1, RANTES and KC, in M-Mcpip1^−/−^ mice compared to Mcpip1^fl/fl^ controls. Additionally, the levels of MIP-1α, MIP-1β, GM-CSF, G-CSF, IL-3, IL-5, IL-12p70 and IL-13 were also significantly greater in M-Mcpip1^−/−^ mice than in Mcpip1^fl/fl^ controls inoculated with LPS (Supplementary Figure S1). Moreover, without LPS challenge, there was essentially no inflammation in the lungs of 2-month-old Mcpip1^fl/fl^ mice, and relatively minor inflammation was observed in the lungs of M-Mcpip1^−/−^ mice. With LPS challenge, pulmonary inflammation in both mouse strains was enhanced, but was much more severe in M-Mcpip1^−/−^ mice compared to Mcpip1^fl/fl^ mice (Figure 2b). Inflammatory cells observed in the lungs of M-Mcpip1^−/−^ mice were primarily macrophages and neutrophils, as shown by staining with anti-F4/80 and anti-Gr-1 antibodies (Figure 2c). Activated macrophages are known to secrete inflammatory cytokines, reactive oxygen species and enzymes, which can have damaging effects on epithelial-endothelial barriers in the lungs, leading to pulmonary edema, an important component of the severe form of acute lung injury, acute respiratory distress syndrome.^26^ Pulmonary edema, measured as a significant increase in the wet-to-dry lung weight ratio, was observed in both strains with LPS challenge, but was much more severe in M-Mcpip1^−/−^ mice than in Mcpip1^fl/fl^ controls (Figure 2d). Furthermore, we measured the mRNA levels of inflammatory markers for macrophage activation in the lungs of both strains. As shown in Supplementary Figure S2, without LPS challenge, the mRNA levels of CXCL1, CXCL2, CCL4, IL-6, MCP1, IL-1β, and CXCL13 were higher in the lungs of M-Mcpip1^−/−^ mice than in Mcpip1^fl/fl^ controls. After LPS challenge, the mRNA levels for these markers increased to a much greater extent in the lungs of M-Mcpip1^−/−^ mice compared to Mcpip1^fl/fl^ controls. By contrast, the anti-inflammatory cytokine IL-10 was decreased in LPS-challenged M-Mcpip1^−/−^ mice. The survival rate within the time window was closely observed, and all mice from both genotypes survived under this condition. Thus, deletion of MCPIP1 in myeloid cells rendered mice highly susceptible to LPS-induced inflammation and lung injury.

### Identification of C/EBPβ and C/EBPδ mRNAs as targets of MCPIP1 RNase in BMDMs

**Figure 3:**
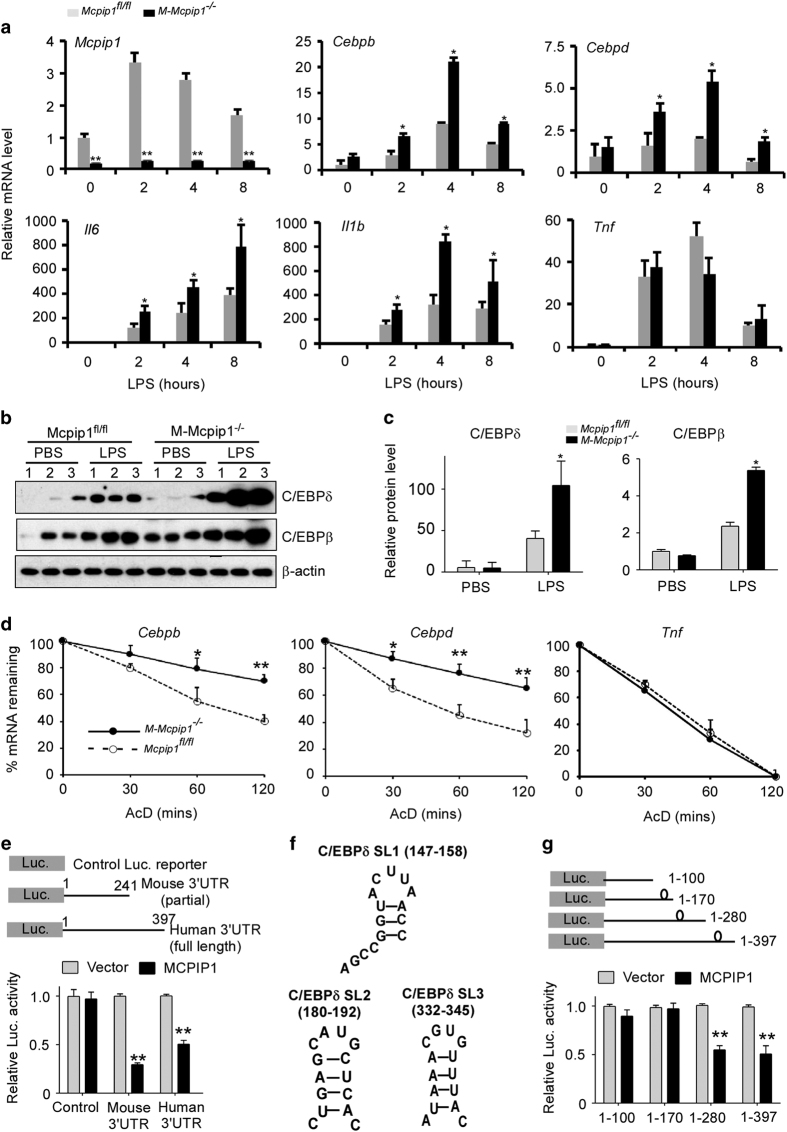
Identification of C/EBPβ and C/EBPδ mRNAs as the targets of MCPIP1 RNase. (**a**) qPCR analysis of the mRNA levels for MCPIP1, C/EBPβ, C/EBPδ, and other inflammatory cytokines in BMDMs from Mcpip1^flfl^ and M-Mcpip1^−/−^ mice treated with LPS for the indicated times. Data are representative of three independent experiments with 3 mice per group. *P<0.05 by Student’s *t*-test. (**b**) Immunoblot analysis of the C/EBPβ and C/EBPδ protein levels in the lungs of Mcpip1^flfl^ and M-Mcpip1^−/−^ mice treated with PBS or LPS for 8 h. β-actin served as a loading control. (**c**) Fold-change of C/EBPβ and C/EBPδ proteins in experiments presented in (**b**) were determined by densitometry and normalized to β-actin. **P<0.01, *P<0.05 by Student’s *t*-test. Data are representative of three independent experiments. (**d**) BMDMs from Mcpip1^flfl^ and M-Mcpip1^−/−^ mice were stimulated with LPS (1 μg/ml) for 4 h and then incubated with AcD (5 μg/ml) for different times as indicated. The mRNA levels were measured by qPCR and normalized to the actin mRNA levels. Data were presented as the means±s.d., *n*=4. (**e**) The reporter of Luc-Control or Luc.-mouse C/EBPδ 3′UTR or Luc.-human C/EBPδ 3′UTR was transfected with or without Flag-MCPIP1 into Raw264.7 cells. Twenty-four hours later, cells were collected for analysis of luciferase activity and normalized to control renilla activity. *N*=4, *P<0.05. **P<0.01 versus vector. Data are representative of three independent experiments. (**f**) The stem-loop structures on human C/EBPδ 3′UTR identified by mfold. (**g**) The serial deletion reporters, as indicated, were transfected with or without Flag-MCPIP1 into Raw264.7 cells. Twenty-four hours later, cells were collected for analysis of luciferase activity and normalized to control renilla activity. *N*=4, **P<0.01 versus vector. Data are representative of three independent experiments.

To explore the mechanisms by which a deficiency of MCPIP1 in macrophages bolsters the response to LPS, the mRNA levels of several inflammatory markers were measured in BMDMs stimulated with LPS (1 μg/ml) for 0 to 8 h (Figure 3a). First, we confirmed that the MCPIP1 mRNA levels were extremely low to non-detectable in LPS-stimulated BMDMs from M-MCPIP1^−/−^ mice. IL-6 and IL-1β mRNAs, two known targets of MCPIP1 RNase,^21,22^ were significantly increased in LPS-stimulated BMDMs from M-Mcpip1^−/−^ versus control mice. The TNFα mRNA levels were not significantly different in LPS-stimulated BMDMs from M-Mcpip1^−/−^ compared to controls, and it is known that TNFα mRNA is not a direct target of MCPIP1 RNase.^22^ Interestingly, mRNAs for both C/EBPβ and C/EBPδ were also significantly increased in LPS-stimulated BMDMs from M-Mcpip1^−/−^ mice compared to controls (Figure 3a). Immunoblot analysis further confirmed that the protein levels of C/EBPβ and C/EBPδ were higher in LPS-stimulated lungs from M-Mcpip1^−/−^ mice than Mcpip1^fl/fl^ controls (Figures 3b and c). These intriguing results suggest that C/EBPβ and C/EBPδ mRNAs may be direct targets of MCPIP1. To further examine whether the MCPIP1 deficiency affects mRNA degradation of C/EBPβ and C/EBPδ, BMDMs were first treated with LPS for 4 h and then incubated with actinomycin D (AcD, 5 μg/ml) for different times, as indicated in Figure 3d. qPCR analysis showed that the mRNA levels of C/EBPβ and C/EBPδ were more stabilized in the cells from M-Mcpip1^−/−^ mice than from Mcpip1^fl/fl^ mice (Figure 3d). As a negative control, the decay of TNF mRNA was not altered in cells. It was recently reported that C/EBPβ mRNA is a direct target of MCPIP1 RNase.^27^ Our results further confirm that C/EBPβ is also regulated in macrophages. It has previously been reported that MCPIP1 RNase targets the 3′ UTR of its mRNA substrates, thereby promoting degradation.^22^ We therefore focused on studying the C/EBPδ 3′UTR. To examine whether MCPIP1 promotes degradation of C/EBPδ mRNA by targeting its 3′UTR, luciferase reporters containing the mouse C/EBPδ 3′UTR (1–241) (GeneCopoeia) and human C/EBPδ 3′UTR (1–397), as well as control reporters, were co-transfected with an empty vector or Flag-MCPIP1 in Raw264.7 cells by electroporation (Amaxa). As shown in Figure 3e, both reporters containing the C/EBPδ 3′UTR were significantly suppressed by overexpression of MCPIP1. As previously reported, MCPIP1 preferentially binds to stem-loop-like structures.^14^ We identified three stem-loops on human C/EBPδ 3′UTR by mfold (Figure 3f). Through serial deletion, we further defined the second stem-loop (180–192) as the putative binding site of MCPIP1 (Figure 3g). As both C/EBPβ and C/EBPδ are critical regulators of proinflammatory cytokines and chemokines,^28,29^ it appears that one mechanism by which myeloid MCPIP1 suppresses LPS-induced inflammation and lung injury is to promote C/EBPβ and C/EBPδ mRNA degradation.

### Pharmacological inhibition of MALT1 protease activity selectively increased the MCPIP1 protein levels in macrophages and inhibited macrophage activation

**Figure 4:**
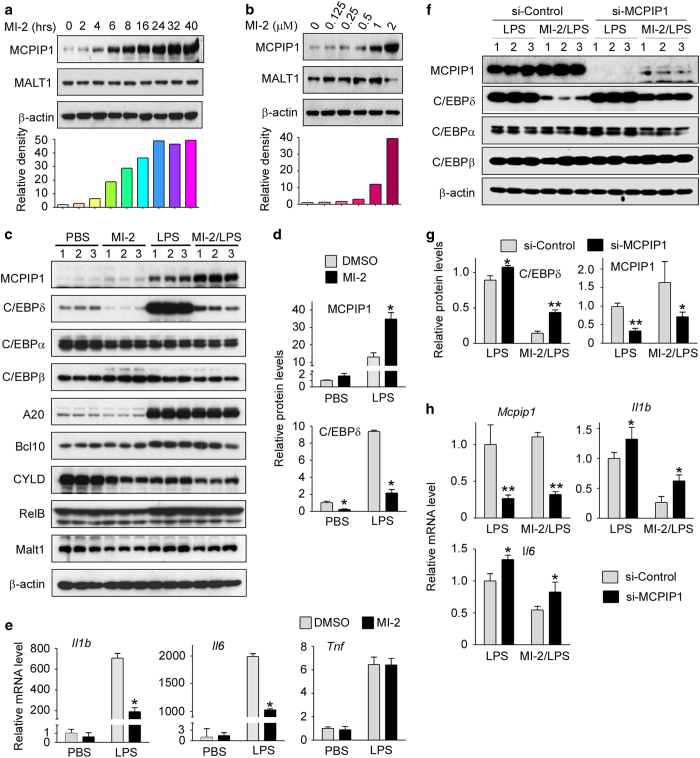
Pharmacological inhibition of MALT1 with MI-2 selectively increased the abundance of the MCPIP1 protein and suppressed LPS-induced macrophage activation. (**a**, **b**) Raw264.7 cells were treated with MI-2 for different durations or doses as indicated. Cell lysates were subjected to analysis by immunoblot with the MCPIP1 antibody. β-actin served as a loading control. Fold changes of the MCPIP1 protein levels were determined by densitometry and normalized to β-actin. Data are representative of three independent experiments. (**c**) Raw264.7 were pretreated with or without 1 μM MI-2 for 1 h and then stimulated with or without 1 μg/ml LPS for 4 h. The protein levels from different genes were determined by Western blot with individual antibodies as indicated. β-actin served as a loading control. (**d**) Fold-change of the MCPIP1 and C/EBPδ protein levels were determined by densitometry and normalized to β-actin. Data are presented as the means±s.d. (*n*=3), *P<0.05 by Student’s *t*-test. (**e**) Raw264.7 were pretreated with or without 1 μM of MI-2 for 30 min and then stimulated with or without 1 μg/ml LPS for 4 h. The mRNA levels of selected cytokines were determined by qPCR analysis and normalized to the β-actin mRNA levels. Data are presented as the means±s.d. (*n*=4), *P<0.05 by Student’s *t*-test. (**f**) Raw264.7 cells were transfected with si-Control or si-MCPIP1 for 24 h. The transfected cells were pretreated with or without 1 μM MI-2 for 1 h and then stimulated with or without 1 μg/ml of LPS for 4 h. The protein levels of different genes were determined by immunoblot with specific antibodies. β-actin served as a loading control. (**g**) Fold-change of the MCPIP1 and C/EBPδ protein levels were determined by densitometry and normalized to β-actin. Data are presented as the means±s.d. (*n*=3), *P<0.05 by Student’s *t*-test. (**h**) Raw264.7 cells were transfected with si-Control or si-MCPIP1 for 24 h. Transfected cells were pretreated with or without 1 μM MI-2 for 1 h and then stimulated with or without 1 μg/ml LPS for 4 h. The mRNA levels of MCPIP1 and selected cytokines were determined by qPCR analysis and normalized to actin mRNA levels. Data are presented as the means±s.d. (*n*=4), *P<0.05 by Student’s *t*-test.

It has previously been reported that MALT1 protease cleaves MCPIP1 in T cells.^30^ However, it is currently unknown whether MALT1 protease also cleaves MCPIP1 in macrophages. MI-2 is a specific inhibitor of MALT1 protease activity.^31^ To examine whether pharmacological inhibition of MALT1 protease activity by MI-2 enhanced the MCPIP1 protein levels in macrophages, Raw264.7 cells, a murine macrophage cell line, were treated with different doses of MI-2 for 8 h or with 1 μM MI-2 for different lengths of time. As shown in Figures 4a and b, MI-2 significantly increased the MCPIP1 protein levels in macrophages in time- and dose-dependent manners. As previously reported, MALT1 protease targets several signal regulators, such as A20, CYLD, BCL10 and RelB.^32–35^ To examine whether MI-2 also enhanced these protein levels, we incubated macrophages with or without 1 μM MI-2 for 1 h and then stimulated the cells with 1 μg/ml LPS for 4 h. The cell lysates were analyzed by immunoblot with different antibodies, as indicated in Figure 4c. As shown in Figure 4c, MALT1 is constitutively expressed in macrophages, and its expression was not affected by treatment with MI-2 and/or LPS. Increased expression of A20, BCL10, and RelB was induced by LPS, but not affected by MI-2 treatment. CYLD expression was not affected by LPS or MI-2 treatment. MCPIP1 expression was increased by MI-2 treatment, and this effect was more dramatic in LPS-stimulated cells (Figures 4c and d). By contrast, the C/EBPδ levels were decreased by MI-2 treatment, and this effect was particularly dramatic in LPS-stimulated cells (Figures 4c and d). By contrast, the C/EBPα and C/EBPβ protein levels were not significantly altered by either MI-2 or LPS treatment. These results suggest that MI-2 selectively enhanced the MCPIP1 protein levels in LPS-stimulated macrophages by preventing MALT1-mediated degradation of MCPIP1. These results also suggest that enhanced MCPIP1 levels, due to inhibition of MALT1, result in the enhanced degradation of C/EBPδ,

To examine whether MI-2 suppressed LPS-induced macrophage activation, Raw264.7 cells were pretreated with or without 1 μM MI-2 for 1 h and then stimulated with 1 μg/ml LPS for 4 h. The mRNA levels of macrophage activation markers were then analyzed by qPCR. As shown in Figure 4e, the mRNA levels of TNFα, IL-1β and IL-6 were significantly increased by LPS stimulation, as expected. Induction of IL-1β and IL-6, but not TNFα mRNA expression, was dramatically inhibited by MI-2 treatment. These observations are consistent with the conclusion that MCPIP1 degrades IL-1β and IL-6, but not TNFα mRNA, and that inhibition of MALT1 with MI-2 bolsters the MCPIP1 levels, resulting in enhanced degradation of IL-1β and IL-6 mRNA.

To confirm that MI-2-mediated inhibition of macrophage activation is dependent on MCPIP1 expression, siRNA-control and siRNA-MCPIP1 were transfected into macrophages. After 24 h, transfected cells were treated with or without MI-2 and then stimulated with or without LPS. As shown in Figures 4f–h, both the MCPIP1 protein and mRNA levels were efficiently reduced by siRNA treatment. MI-2-mediated inhibition of C/EBPδ protein expression was severely compromised by siMCPIP1 treatment. Furthermore, MI-2-mediated inhibition of IL-1β and IL-6 mRNA expression was compromised by siMCPIP1 treatment. These results collectively suggest that inhibition of MALT1 activity with MI-2 results in increased MCPIP1 levels in macrophages, which significantly inhibits macrophage inflammation by decreasing the expression levels of IL-1β, IL-6, and C/EBPδ. Similar results were also observed in THP1-derived macrophages, a human macrophage cell line (Supplementary Figure S3), and human primary monocytes (Supplementary Figure S4).

### SiRNA-mediated knockdown of MALT1 also selectively increased the MCPIP1 protein levels in macrophages and inhibited macrophage activation

**Figure 5:**
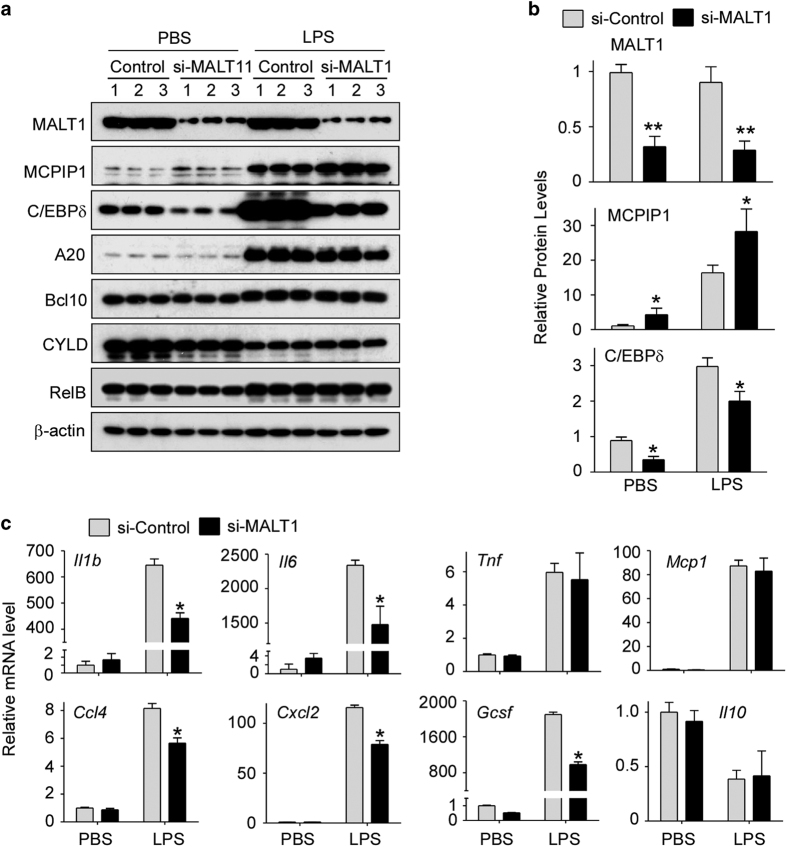
SiRNA-mediated knockdown of MALT1 selectively increased the abundance of the MCPIP1 protein and suppressed LPS-induced macrophage activation. (**a**) Raw264.7 cells were transfected with si-Control or si-MALT1 for 24 h. Transfected cells were stimulated with or without 100 ng/ml LPS for 8 h. The protein levels of different genes were determined by immunoblot with individual antibodies as indicated. β-actin served as a loading control. (**b**) Fold-change of the MCPIP1 and C/EBPδ protein levels were determined by densitometry and normalized to β-actin. Data are presented as the means±s.d. (*n*=3), *P<0.05 by Student’s *t*-test. (**c**) Raw264.7 cells were transfected with si-Control or si-MALT1 for 24 h. Transfected cells were stimulated with or without 100 ng/ml LPS for 8 h. The mRNA levels of MCPIP1 and cytokines as indicated were determined by qPCR analysis and normalized to actin mRNA levels. Data are presented as the means±s.d. (*n*=4), *P<0.05 by Student’s *t*-test.

To exclude any non-specific effects of MI-2 on the MCPIP1 protein levels in macrophages, Raw264.7 cells were transfected with siRNA targeting MALT1 or control siRNA. After 24 h, transfected cells were treated with or without LPS for 4 h. The cell lysates were collected for immunoblot analysis. As shown in Figures 5a and b, the MALT1 protein levels were efficiently reduced by si-MALT1 by 80% compared to si-Control-treated cells. Consistently, the MCPIP1 protein levels were increased by si-MALT1 in resting macrophages and further increased in LPS-treated cells. By contrast, the C/EBPδ protein levels were significantly decreased by si-MALT1. Interestingly, previously reported targets of MALT1, including A20, BCL10, CYLD and RelB, were not affected by si-MALT1 treatment in macrophages. These results suggest that MALT1 protease selectively cleaved MCPIP1 both in resting and activated macrophages, but did not cleave other targets that have been identified in lymphocytes, such as A20, BCL10, CYLD and RelB. The mechanisms underlying the discrimination between the two types of cell lineages remain to be further investigated. To examine whether siRNA-mediated knockdown of MALT1 suppresses LPS-induced macrophage activation, Raw264.7 cells were transfected with siRNA for MALT1 or control siRNA. After 24 h, transfected cells were treated with or without LPS for 4 h, and the mRNA levels of the macrophage activation markers were analyzed by qPCR. As shown in Figure 5c, the mRNA levels of IL-1β, IL-6 CCL4, CXCL2 and G-CSF were significantly increased by LPS stimulation, as expected, and these levels were dramatically suppressed by si-MALT1 treatment. However, the mRNA levels of TNFα and MCP1 were induced by LPS, but were not affected by siMALT1 treatment. The IL-10 mRNA levels were decreased by LPS but were not affected by siMALT1 treatment (Figure 5c). These results demonstrate that reducing the MALT1 levels by siRNA consistently enhances the MCPIP1 protein levels in macrophages, significantly inhibiting macrophage inflammatory signaling by decreasing expression of IL-1β, IL-6, and C/EBPδ.

### MI-2 administration prevented LPS-induced MCPIP1 protein degradation in the lungs

**Figure 6:**
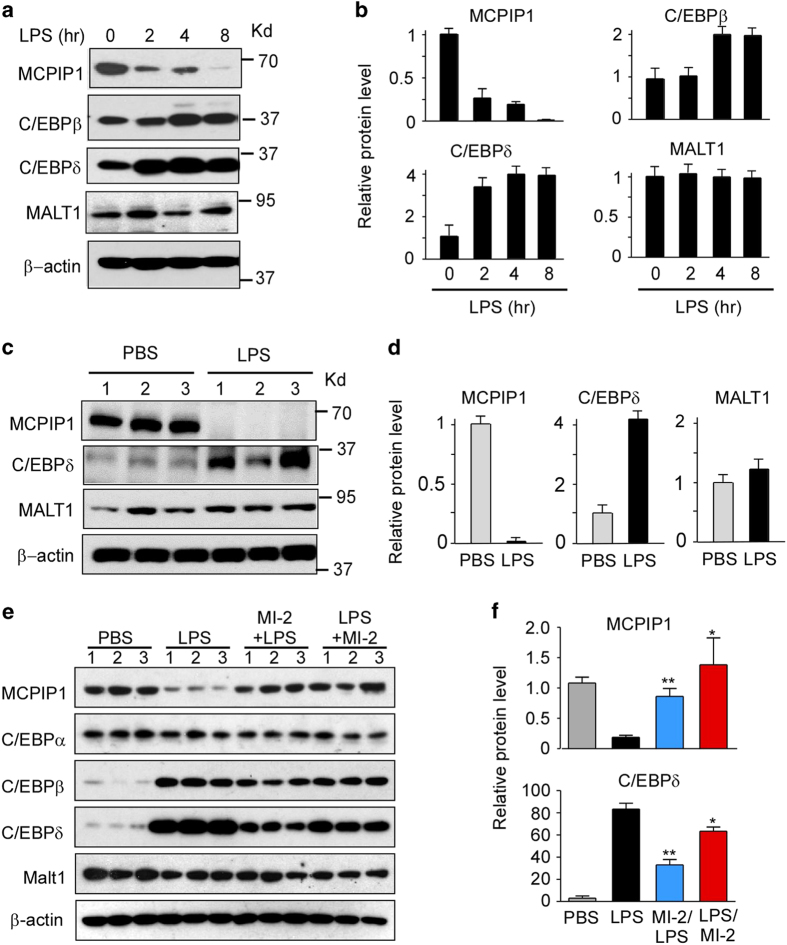
Administration of MI-2 prevented LPS-induced MCPIP1 protein degradation *in vivo*. (**a**) Adult C57BL/6 mice were intraperitoneally injected with LPS (25 mg/kg body weight) for different times as indicated. The mice were euthanized, and the lungs were collected for immunoblot analysis with different antibodies, as indicated. β-actin serves as a loading control. (**b**) Fold-change of the protein levels were determined by densitometry and normalized to β-actin. Data are presented as the means±s.d. (*n*=3). (**c**) Adult C57BL/6 mice were intraperitoneally injected with LPS (25 mg/kg body weight) for 8 h. Mice were euthanized, and the lungs were collected for immunoblot analysis with specific antibodies, as indicated. β-actin serves as a loading control. (**d**) Fold-changes of the protein levels were determined by densitometry and normalized to β-actin. Data are presented as the means±s.d. (*n*=3). (**e**) Adult C57BL/6 mice were intraperitoneally injected with PBS or LPS (25 mg/kg body weight) for 8 h. One group of mice was pretreated with MI-2 for 2 h and then injected with LPS for 8 h; the other group of mice was injected with LPS for 2 h and then treated with MI-2. Mice were euthanized 8 h after LPS injection. The lungs were collected for immunoblot analysis with specific antibodies as indicated. β-actin serves as a loading control. (**f**) Fold-changes of protein levels in (**e**) were determined by densitometry and normalized to β-actin. Data are presented as the means±s.d. (*n*=3). *P<0.05, **P<0.01 by Student’s *t*-test.

As previously reported, MCPIP1 is subjected to phosphorylation and degradation at early time points during LPS and cytokine-induced macrophage activation.^36^ However, changes in the MCPIP1 protein levels *in vivo* during LPS-induced systemic inflammation and lung injury remain unknown. We examined the MCPIP1 protein levels in mouse lungs 0, 2, 4 and 8 h after LPS injection (25 mg/kg body weight, i.p.) by immunoblot analysis. As shown in Figures 6a and b, the MCPIP1 protein levels gradually decreased after LPS treatment and were essentially undetectable by 8 h. The MALT1 protein levels were not changed by LPS administration, whereas the C/EBPδ and C/EBPβ protein levels were increased 2 and 4 h after LPS administration and remained elevated at 8 h. To further verify that LPS-treatment promoted MCPIP1 protein degradation in the lungs, mice were injected with LPS (25 mg/kg body weight, i.p.) and sacrificed after 8 h. Immunoblot analysis showed that the MCPIP1 protein levels were markedly decreased in lungs from LPS-treated mice compared with those from control mice, whereas the C/EBPδ protein levels were significantly increased (Figures 6c and d). These results suggest that LPS administration may induce degradation of MCPIP1 *in vivo*. To test whether administration of MI-2 could prevent LPS-induced MCPIP1 protein degradation *in vivo*, mice were treated with or without MI-2 (25 mg/body weight, i.p.) and then treated with or without LPS (25 mg/kg body weight, i.p.) 2 h later. Another group of mice was injected with LPS and then treated with MI-2 2 h later. All mice were sacrificed 8 h after LPS injection, and the lungs were collected for immunoblot analysis. As shown in Figures 6e and f, LPS induced MCPIP1 degradation in the lungs as expected. MI-2 treatment either before or after LPS injection prevented LPS-induced decreases in the MCPIP1 protein levels. While he MALT1 and C/EBPα protein levels were not changed by any of these treatments, the C/EBPβ and C/EBPδ protein levels were both increased by LPS and decreased by MI-2 treatment. Taken together, these results demonstrate that the MCPIP1 protein levels in the lungs are preserved by pharmacological inhibition of MALT1 protease, suggesting that MALT1 mediates LPS-induced degradation of MCPIP1 protein in the lungs. Furthermore, pharmacologic inhibition of MCPIP1 degradation with MI-2 results in increased levels of C/EBPβ and C/EBPδ proteins in the lungs.

### Administration of MI-2 protects mice from LPS-induced inflammation, lung injury and death

**Figure 7:**
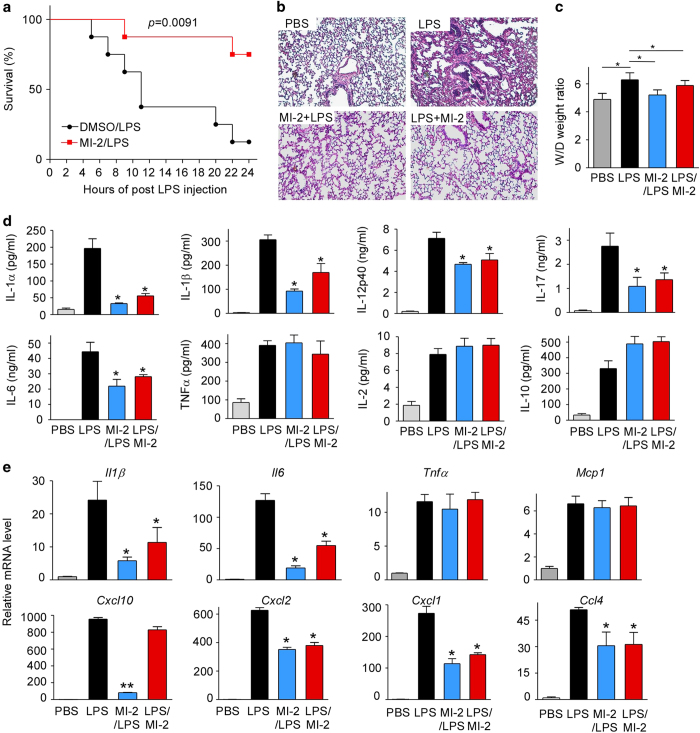
Administration of MI-2 protected mice from LPS-induced inflammation, lung injury and death. (**a**) C57BL/6 mice from Jackson Laboratory were divided into two groups: one group was only injected with a lethal dose of LPS (40 mg/kg body weight, i.p.); the other group was pre-treated with MI-2 (25 mg/Kg body weight, i.p.) at 2 h before LPS injection and another dose at 2 h post-LPS injection. All mice were closely monitored, and ‘moribund status’ was equated with death to minimize the discomfort of the mice. The survival rate was recorded. The data were analyzed by the log-rank Mantel-Cox test. P=0.0091, *N*=8. (**b** and **d**) Adult C57BL/6 mice were divided into four groups. Two groups of mice were intraperitoneally injected with PBS or LPS (25 mg/kg body weight) for 8 h. The third group of mice was pretreated with MI-2 for 2 h and then injected with LPS for 8 h; the fourth group of mice was injected with LPS for 2 h and then treated with MI-2. Mice were euthanized 8 h after LPS injection. Sera and lungs were collected for ELISA, H&E staining or qPCR analysis. The serum cytokine levels were measured by ELISA (**d**), and lung sections were analyzed by H&E staining (**b**). Data are presented as the means±s.d. (*n*=3). *P<0.05 by Student’s *t*-test. Data are representative of three independent experiments. (**c**) Changes of lung wet-to-dry weight ratios in mice. Data are presented as the means±s.d. (*n*=3), *P<0.05 by Student’s *t*-test. (**e**) qPCR analysis of the mRNA levels for selected cytokines and chemokines in the lungs collected from the mice in (**b**). Data are representative of three independent experiments with 3 mice per group. *P<0.05 by Student’s *t*-test.

To test whether administration of MI-2 protects mice from LPS-induced death, mice were divided into two groups: one group was injected solely with a lethal dose of LPS (40 mg/kg body weight, i.p.); the other group was pre-treated with MI-2 (25 mg/Kg body weight, i.p.) 2 h before LPS injection and another dose at 2 h post-LPS injection. All mice were closely monitored, and a ‘moribund status’ was equated to death to minimize the discomfort of the mice. The survival rate was recorded. As shown in Figure 7a, administration of MI-2 significantly improved survival of mice injected with LPS. To test whether administration of MI-2 protects mice from LPS-induced inflammation and lung injury, mice were divided into four groups as described in Figure 6e. All mice were sacrificed 8 h after LPS injection. Sera and lungs were collected for analysis by ELISA, histology and qPCR. LPS inoculation induced inflammatory cell infiltration and lung edema in the lungs, as expected (Figures 7b and c). Intriguingly, MI-2 treatment, either before or after LPS injection, significantly attenuated LPS-induced lung injury, as demonstrated by decreased inflammatory cell infiltrates and edema (Figures 7b and c). LPS inoculation induced significant increases in the serum concentrations of IL-1α, IL-1β, IL-6, IL-12p40, TNFα, IL-17, IL-2 and IL-10. MI-2 treatment, either before or after LPS inoculation; significantly attenuated the response to LPS for IL-1α, IL-1β, IL-6, IL-12p40, and IL-17; but did not affect the production of TNFα, IL-2, and IL-10 (Figure 7d). Additional cytokine levels, such as for IL-2, IL-5, IL-9, Il-13, MIP-1α, MIP-1β, Eotaxin, and G-CSF, were not affected by MI-2 treatment, except that the level of IFNγ was decreased by MI-2 treatment (Supplementary Figure S5). Finally, we measured the mRNA levels of inflammatory markers for macrophage activation in the lungs of these mice. As shown in Figure 7e, the mRNA levels of IL-1β, IL-6, TNFα, MCP1, CXCL10, CXCL2, CXCL1, and CCL4 were increased by LPS injection. MI-2 treatment, either before or after LPS injection, attenuated LPS-induced mRNA expression of IL-1β, IL-6, CXCL2, CXCL1, and CCL4. MI-2 treatment did not suppress LPS-induced expression of TNFα and MCP1 mRNA in the lungs and only suppressed the CXCL10 levels when it was given prior to LPS treatment. Taken together, these results suggest that pharmacological inhibition of MALT1 protease activity with MI-2 protects mice from LPS-induced inflammation, lung injury and death.

## Discussion

The major new findings of the current study were: 1) that MCPIP1 expression in myeloid cells plays a central role in protecting against LPS-induced inflammation, lung injury and death; 2) that decreases in the MCPIP1 levels in macrophages following LPS inoculation are attributable, at least in part, to increased proteolysis by MALT1; 3) that pharmacologic inhibition of MALT1 with MI-2 protects against LPS-induced lung injury; 4) that both C/EBPβ and C/EBPδ mRNAs are targets of MCPIP1 RNase; and 5) that the severe inflammatory syndrome that develops in mice with a global MCPIP1 deficiency is significantly delayed and has decreased severity in mice with a myeloid-specific MCPIP1 deficiency.

The conclusions stated above are based on the following experimental evidence. First, we demonstrated that inflammatory damage to the lungs and that the protein and mRNA levels of various proinflammatory cytokines were elevated in the serum and lungs, respectively, of LPS-treated myeloid-specific MCPIP1 knockout mice compared to LPS treated control mice. We also demonstrated that mRNA for IL-6, IL-1β, C/EBPβ and C/EBPδ and that the protein levels for C/EBPβ and C/EBPδ were higher in LPS-stimulated BMDM’s from myeloid specific MCPIP1 knockout mice compared to BMDMs from LPS-treated control mice. Additionally, MCPIP1 overexpression in the Raw264.7 murine macrophage cell line resulted in suppressed mRNA levels for C/EBPβ and C/EBPδ. Next, we demonstrated that the MCPIP1 protein levels in Raw264.7 cells were increased by treatment with MI-2, a specific inhibitor of MALT1 protease, in a time- and dose-dependent manner. Furthermore, MI-2 treatment of LPS-stimulated RAW264.7 cells enhanced the MCPIP1 protein levels and decreased the C/EBPδ protein and IL-1β and IL-6 mRNA levels. That the effects of MI-2-mediated inhibition of MALT1 were mediated through MCPIP1 and were confirmed by demonstrating that MCPIP1 siRNA reversed the effects of MI-2 treatment. We also demonstrated that treatment of macrophages with MALT1 siRNA consistently enhanced the MCPIP1 protein levels and decreased expression of IL-1β and IL-6 mRNAs as well as the C/EBPδ protein. Finally, we demonstrated that MI-2 treatment increased the MCPIP1 protein levels in the lungs as well as decreased inflammatory infiltrates and the C/EBPβ and C/EBPδ protein levels, along with protein and mRNA for several other proinflammatory cytokines, in the lungs of LPS-injected mice. These collective findings support the conclusions that myeloid MCPIP1 suppresses production of C/EBPβ, C/EBPδ, and a variety of proinflammatory cytokines through its RNase activity and that pharmacologic inhibition of MALT1 protease enhances the MCPIP1 protein levels, thereby suppressing LPS-induced inflammatory responses. The rationale for assessing C/EBPβ and C/EBPδ rather other transcriptional factors in M-Mcpip1^−/−^ BMDMs is that C/EBPβ and C/EBPδ have been reported to be the major transcriptional drivers of the inflammation and lung injury, and many cytokine expression level changes are not due to their mRNA decay. We therefore evaluated whether MCPIP1 RNAse affects those cytokine expression levels by targeting major transcription factors, such as C/EBPβ and C/EBPδ. During the preparation of this manuscript, another group independently identified C/EBPβ as a new target of MCPIP1 RNase. Our results further confirm it as target of MCPIP1 in macrophages.

We also found that a myeloid-specific deficiency of MCPIP1 resulted in no overt phenotypic changes in mice during the first several months of life, in contrast to the early-onset, severe inflammatory syndrome characteristic of the total MCPIP1 deficiency.^22–24^ This suggests that a MCPIP1 deficiency in cell types other than macrophages is also required for the development of the severe inflammatory syndrome phenotype in this strain of mice. It has previously been reported that mice with a T cell-specific MCPIP1 deficiency develop an inflammatory syndrome within 3 months of life,^30^ suggesting that MCPIP1 is indispensable for controlling the activation of both innate and adaptive immune cells.^30^ After 5 months of age, M-Mcpip1^−/−^ mice gradually developed an inflammatory syndrome characterized by splenomegaly, lymphadenopathy, and multi-organ inflammation, which is similar to the phenotypes described for MCPIP1 global knockout mice.^22–24^ The underlying mechanisms by which the myeloid-specific MCPIP1 deficiency contributes to abnormal responses in both innate and adaptive immune systems warrants further investigation.

The extreme sensitivity of M-MCPIP1 KO mice to LPS-induced inflammation and lung injury appears to be attributable, at least in part, to the high serum concentrations of proinflammatory cytokines and chemokines that develop after LPS injection. These, in turn, could be attributed to the high production of inflammatory cytokines by macrophages and other myeloid cells. Expression profiling of inflammatory cytokines in BMDMS showed that many proinflammatory cytokines and chemokines, including MCPIP1 RNase targets and non-targets, were significantly increased in MCPIP1-deficient macrophages. We further identified both C/EBPβ and C/EBPδ mRNAs as targets of MCPIP1 RNase. As C/EBPβ and C/EBPδ are critical transcription factors that drive the expression of a plethora of inflammatory genes and critically contribute to lung inflammatory injuries,^37–40^ enhanced expression of C/EBPβ and C/EBPδ in MCPIP1-deficient macrophages is likely to contribute to the phenotypes observed in M-MCPIP1 KO mice.

In our studies, although MCPIP1 affects both the C/EBPβ and C/EBPβ mRNA expression levels in BMDMs, MI-2 treatment predominantly affects C/EBPδ in macrophages and the lungs, suggesting that the C/EBPδ changes are more robust in Mcpip1-deficient tissues and cells. Indeed, in the transcriptional program of inflammation, C/EBPδ plays a more important role than C/EBPβ.^28^ In addition, the effects of MI-2 on the protein levels of MCPIP1 or C/EBPδ were more dramatic with LPS treatment. LPS stimulation activates the MALT1/BCL10/CARD9 complex and degrades MCPIP1 protein, which may be why the effect of MI-2 is more dramatic with LPS treatment.

Protein degradation of MCPIP1 in activated T cells has been attributed to the protease activity of MALT1,^30^ which is a main component of the signalosome complex CARD11/BCL10/MALT1.^41–43^ In addition to MCPIP1, MALT1 protease also cleaves several other targets, including A20, CYLD, BCL10 and RelB, in lymphocytes.^31–34^ Dong *et al.* reported that the BCL10/MALT1 complex also mediated NF-κB activation in macrophages in response to TLR activation;^44^ however, whether MCPIP1 protein is subjected to cleavage by MALT1 in macrophages has not been previously reported. In the present study, we observed that MI-2, a specific inhibitor of MALT1 protease, markedly increased the MCPIP1 protein levels in both resting and activated macrophages, suggesting that MALT1 protease activity controls the MCPIP1 protein levels under basal and stimulated conditions. Interestingly, other targets of MALT1 identified in lymphocytes, including A20, CYLD, BCL10 and RelB, were not affected in resting or activated macrophages by MI-2 treatment. To further confirm that MALT1 protease-mediated the cleavage of MCPIP1 in macrophages, we used siRNA to knockdown MALT1 expression in macrophages, confirming that MALT1 promoted MCPIP1, but not A20, CYLD, BCL10 or RelB, degradation. The mechanism by which MALT1 protease selectively cleaves MCPIP1 in macrophages, but not other protein targets identified in lymphocytes, remains to be determined. In addition, Iwaski *et al.* reported that LPS induces degradation of MCPIP1 *in vitro* at very early time points.^36^ In our experiments, LPS degraded MCPIP1 in the lungs at 2 h after LPS challenge. We believe that the time course of MCPIP1 degradation *in vivo* and *in vitro* is different. However, we cannot exclude the possibility that other cells in addition to macrophages may also be involved in this process.

Our findings that MI-2 administration prevents MALT1-mediated MCPIP1 degradation *in vivo* and protects mice from LPS-induced inflammation and lung injury, suggests that pharmacological inhibition of MALT1 protease activity by MI-2 may be an effective strategy for preventing/treating sepsis-associated lung injury. It is particularly relevant that MI-2 is not toxic in mice even after prolonged exposure.^31^ Several reports have demonstrated that MI-2 is effective for the prevention/treatment of activated B cell-like diffuse large B cell lymphoma (ABC-DLBCL), HIV and experimental colitis.^31,45,46^ However, several recent studies have shown that mice expressing catalytically inactive MALT1 develop multi-organ inflammation and autoimmunity.^47–50^ The mechanism underlying these results appears to be that MALT1 protease activity is required for the development of Treg cells; disruption of MALT1 protease activity leads to deficient Treg development as well as excessive inflammation and autoimmunity.^47–50^ In our experiments, short-term treatment with MI-2 did not affect expression of IL-10, suggesting that such treatment does not affect Treg differentiation and function. However, further investigation is still required. Furthermore, in light of the essential role of MALT1 in inflammatory cell activation, inhibition of MALT1 protease activity may potentially lead to immune suppression and increased susceptibility to infections, which is another potential problem requiring additional exploration.

Degradation of MCPIP1 occurs through multiple mechanisms in addition to MALT1, such as proteasome-dependent degradation. Indeed, two groups have reported that MG132, an inhibitor of the proteasome, can enhance the MCPIP1 protein levels in vascular smooth muscle cells and skin cells.^51,52^ As the proteasome broadly targets protein degradation, MALT1 inhibitors have significant potential for the treatment of inflammation.

**Figure 8:**
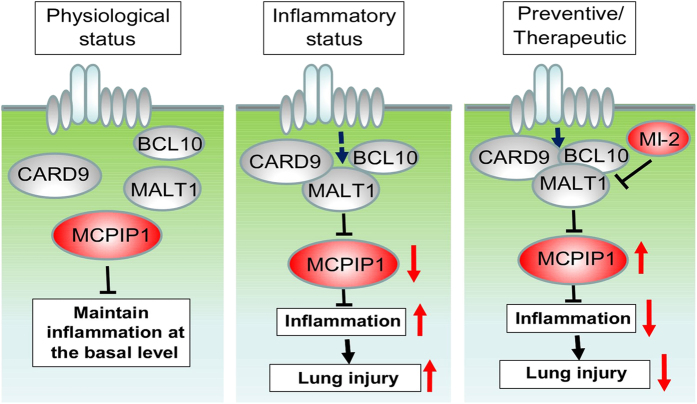
Schematic representation of the central role of MCPIP1 in LPS-induced inflammation and lung injury. As an RNase, MCPIP1 post-transcriptionally controls the production of cytokines and maintains inflammation at a basal level under physiological conditions. In response to inflammatory stimuli (for example, LPS), macrophage CARD9/BCL10/MALT1 signalosome is activated and MALT1 protease mediates MCPIP1 cleavage, which releases the ‘brake’ and initiates the inflammatory response. MI-2 specifically inhibits MALT1 protease activity and preserves the MCPIP1 protein levels, which can prevent/treat LPS-induced inflammation and lung injury.

In summary, the present study is the first to identify the central role of myeloid MCPIP1 in protecting against LPS-induced inflammation, lung injury and death. MCPIP1 is an RNase that contributes to post-transcriptional control of inflammatory cytokine production and helps maintain inflammation at basal levels under physiological conditions.^14^ In response to inflammatory stimuli, the macrophage CARD9/BCL10/MALT1 signalosome is activated and MALT1 protease cleaves MCPIP1, releasing the ‘brake’ that suppresses inflammation and initiating the inflammatory response. MI-2 specifically inhibits MALT1 protease activity and preserves MCPIP1 protein levels, which can prevent/treat LPS-induced inflammation and lung injury (Figure 8).
