# Supplementary material for: Central role of myeloid MCPIP1 in protecting against LPS-induced inflammation and lung injury
Source: Signal Transduct Target Ther. 2017 Dec 8;2:17066–. doi: 10.1038/sigtrans.2017.66 (PMC5721545; doi:10.1038/sigtrans.2017.66)
Supplement: Supplementary Information [file sigtrans201766-s1.doc]

Supplementary Materials

For

“Central role of myeloid MCPIP1 in the protection of LPS-induced inflammation and lung injury”

By Yong Li et al.

Supplementary Figure 1


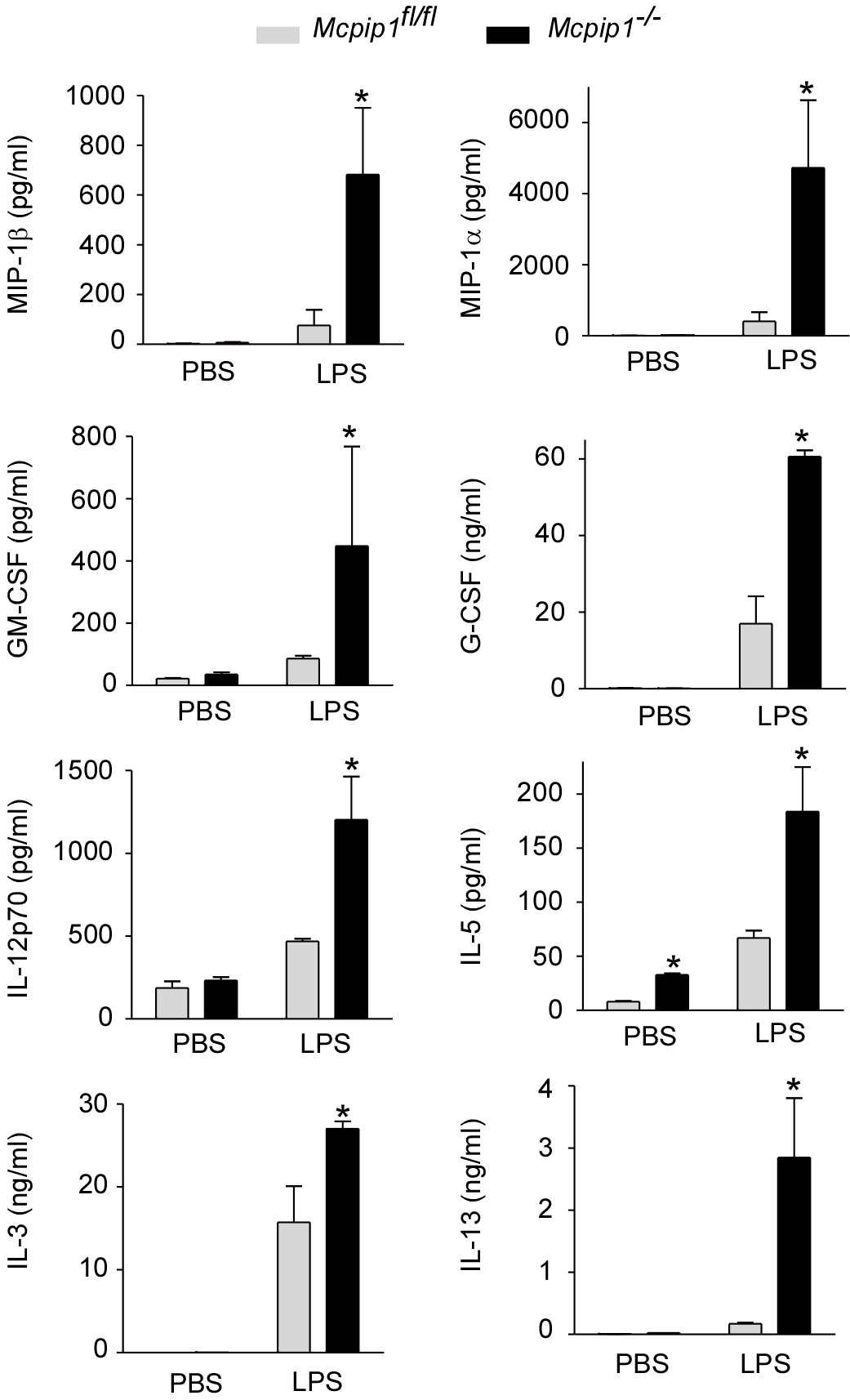


**Supplementary Figure 1. Extended information for Figure 2a**. Serum levels of proinflammatory cytokines and chemokines in Mcpip1^flfl^ and M-Mcpip1^‒/‒^ mice, treated intraperitoneally with PBS or LPS for 8 hours. Data are presented as mean±s.d. (n=5). *P<0.05, vs Mcpip1^flfl^ group.

Supplementary Figure 2


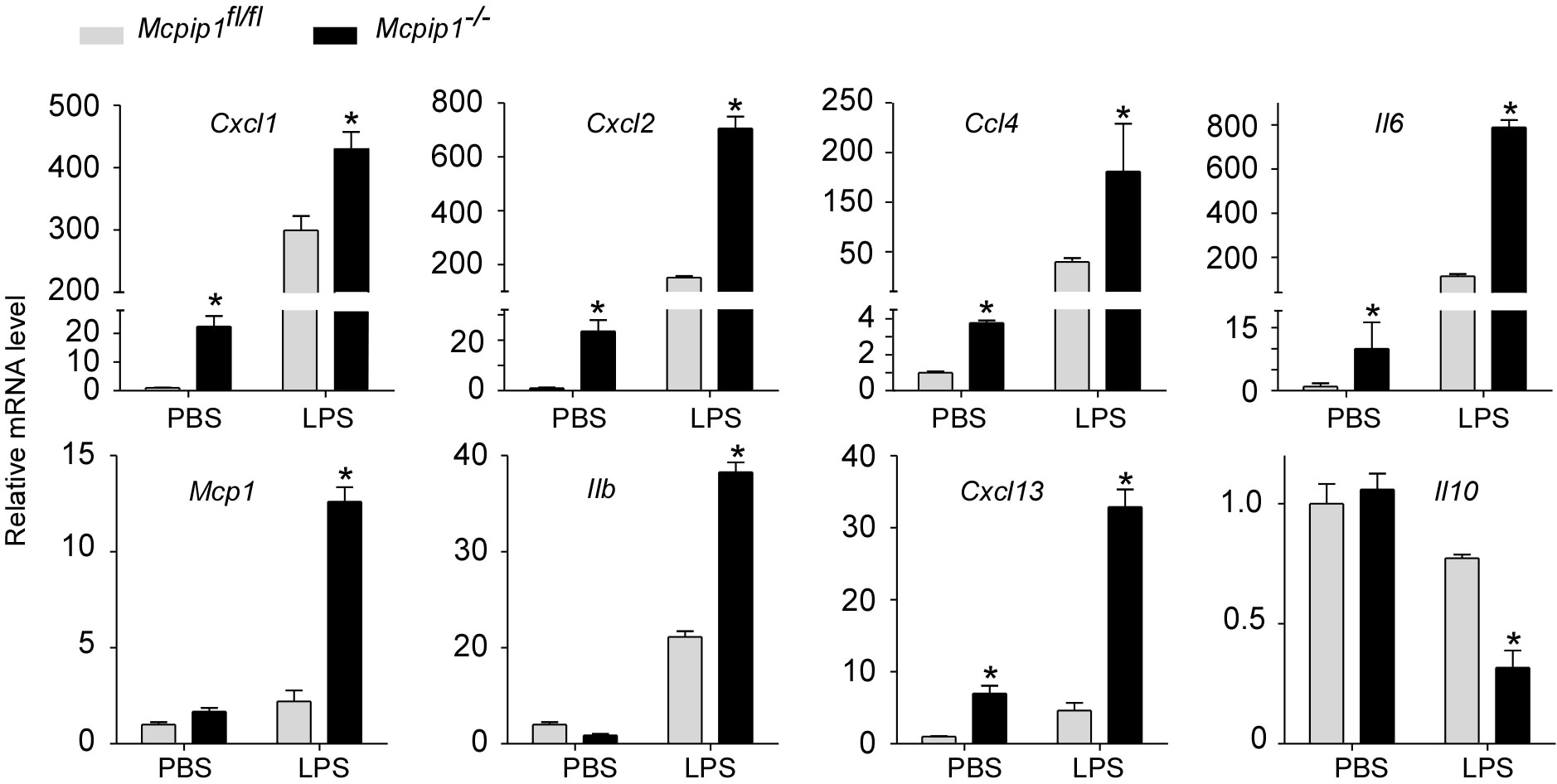


**Supplementary Figure 2. Extended information for Figure 2**. The mRNA levels of proinflammatory cytokines and chemokines in the lungs from Mcpip1^flfl^ and M-Mcpip1^‒/‒^ mice, treated intraperitoneally with PBS or LPS for 8 hours. Data are presented as mean±s.d. (n=5). *P<0.05, vs Mcpip1^flfl^ group.

Supplementary Figure 3


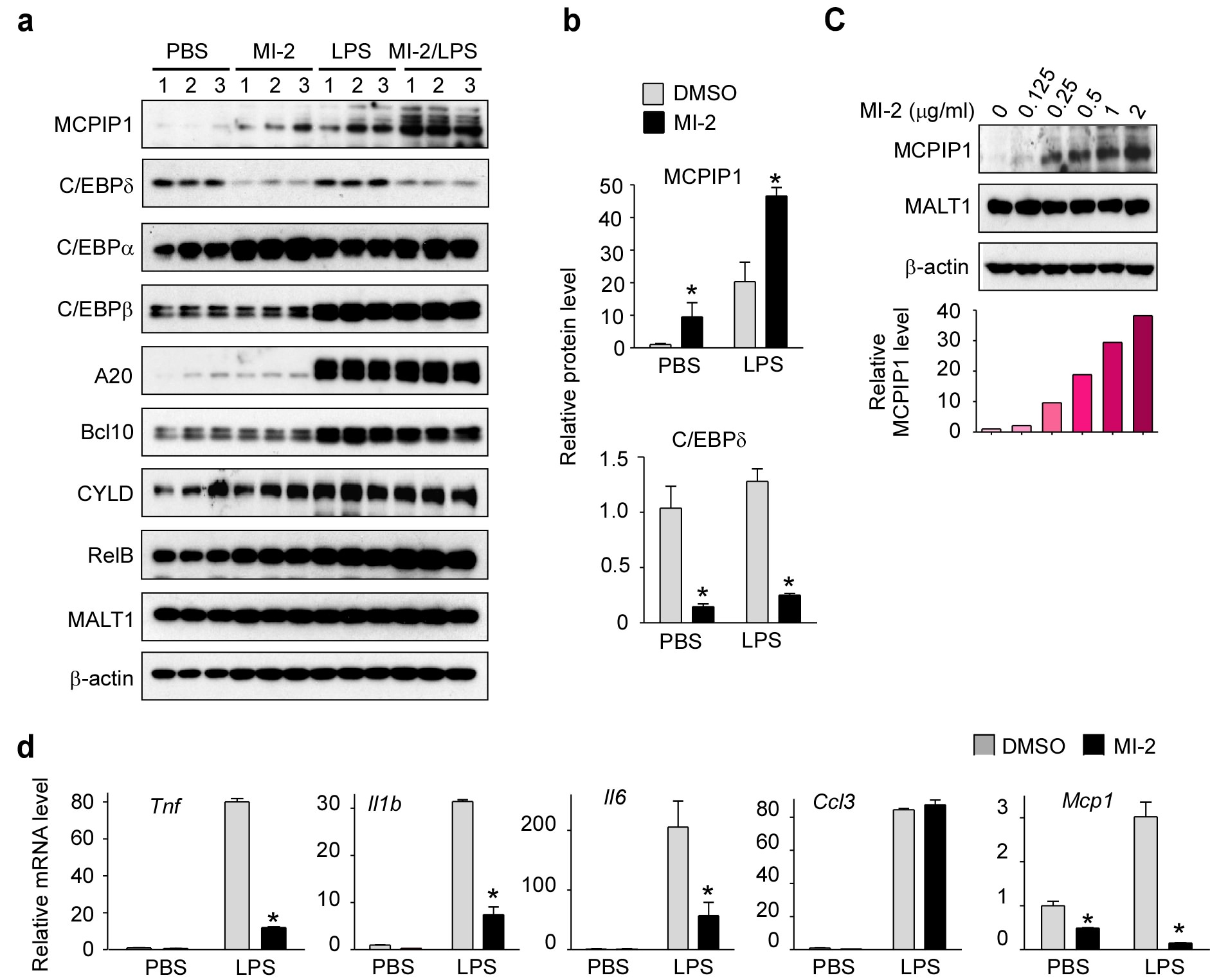


**Supplementary Figure 3.** **Pharmacological inhibition of MALT1 with MI-2 selectively increased the abundance of MCPIP1 protein and suppressed LPS-induced macrophage activation.** (**a**) THP1 cells were incubated with PMA (1 μg/ml) for 7 days. All of THP1 cells differentiated into macrophages. THP1-derived macrophages were pretreated with or without 1 µM MI-2 for 1 hour, then stimulated with or without 1 μg/ml LPS for 4 hours. The protein levels from different genes were determined by western blot with individual antibodies as indicated. β-actin serves as a loading control. (**b**) Fold changes of MCPIP1 and C/EBPδ protein levels were determined by densitometry and normalized to β-actin. Data are presented as mean±s.d. (n=3), *P<0.05 by Student’s t-test. (**c**) THP1-derived macrophages were treated with MI-2 in different doses as indicated. The cell lysates were subjected to analysis by immunoblot with MCPIP1 antibody. β-actin serves as a loading control. Fold changes of MCPIP1 protein levels were determined by densitometry and normalized to β-actin. Data are representative of three independent experiments. (**d**) THP1-derived macrophages were pretreated with or without 1 µM MI-2 for 1 hour, then stimulated with or without 1 μg/ml LPS for 4 hours. The mRNA levels of cytokines as indicated were determined by QPCR analysis and normalized by actin mRNA levels. Data are presented as mean±s.d. (n=4), *P<0.05 by Student’s t-test.

Supplementary Figure 4


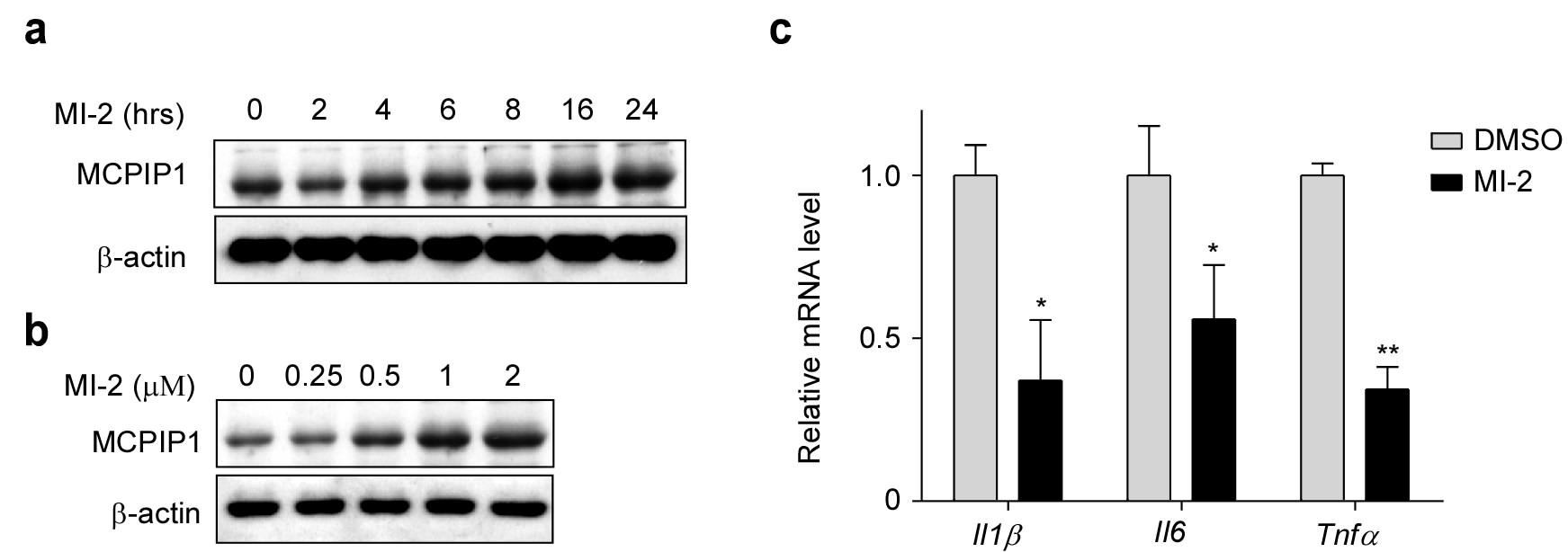


**Supplementary Figure 4.** **MI-2 treatment increased the abundance of MCPIP1 protein and suppressed LPS-induced inflammation in human primary monocytes.** (**a&b**) Human primary monocytes were purchased from Lonza and cultured in the medium provided by the manufacturer. Quiescent cells were treated with MI-2 for indicated times or with different doses as indicated. The cell lysates were subjected to analysis by immunoblot with MCPIP1 antibody. β-actin serves as a loading control. Data are representative of three independent experiments. (**c**) Human primary monocytes were pretreated with or without 1 µM MI-2 for 1 hour, then stimulated with or without 1 μg/ml LPS for 4 hours. The mRNA levels of cytokines as indicated were determined by QPCR analysis and normalized by actin mRNA levels. Data are presented as mean±s.d. (n=4), *P<0.05 by Student’s t-test.

Supplementary Figure 5


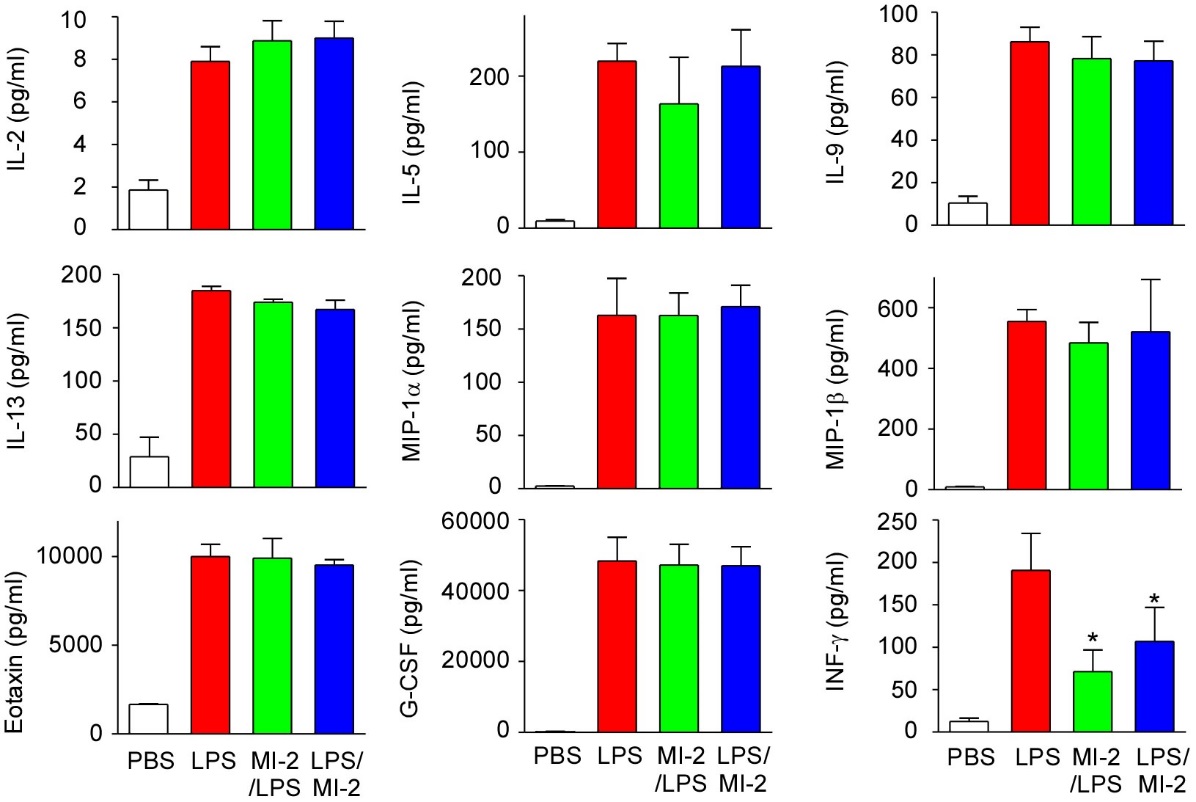


**Supplementary Figure 5. Extended information for Figure 7a**. Serum levels of proinflammatory cytokines and chemokines in the mice, which were divided into four groups. Two groups of mice were intraperitoneally injected with PBS or LPS (25 mg/kg body weight) for 8 hours. The third group of mice were pretreated with MI-2 for 2 hours and then injected with LPS for 8 hours; the fourth group of mice were injected with LPS for 2 hours and then treated with MI-2. The mice were euthanized 8 hours after LPS injection. Serum levels of cytokines were measured by ELISA. Data are presented as mean±s.d. (n=3). *P<0.05 by Student’s t-test. Data were representative of three independent experiments.

Supplementary Table 1: Sequences of QPCR primers used in this study.

| **Primer Name** | **Forward Sequences (5’-3’)** | **Reverse Sequences (5’-3’)** |
| --- | --- | --- |
| Mouse MCPIP1 | CTGCCTCTCAGTCCAGCTCT | GGAGTGAGTCCTGGGTGTGT |
| Mouse C/EBPβ | ACGAGTACAAGATGCGGC | TGAACAAGTTCCGCAGGG |
| Mouse C/EBPδ | CCTGCCATGTACGACGACGAG | GCCGCTTTGTGGTTGCTGTT |
| Mouse IL-1β | AAGGAGAACCAAGCAACGACAAAA | TGGGGAACTCTGCAGACTCAAACT |
| Mouse IL-6 | TCGTGGAAATGAGAAAAGAGTTG | AGTGCATCATCGTTGTTCATACA |
| Mouse TNFα | CTGAGGTCAATCTGCCCAAGTAC | CTGAGGTCAATCTGCCCAAGTAC |
| Mouse MCP1 | TTAACGCCCCACTCACCTGCTG | GCTTCTTTGGGACACCTGCTGC |
| Mouse CCL4 | TCTGTGCAAACCTAACCCCG | GAGGGTCAGAGCCCATTGGT |
| Mouse CXCL1 | CCTGCCCTTATAGGAACAGAAG | AAGCGATGCTCAAACACATTAG |
| Mouse CXCL2 | CCCAGACAGAAGTCATAGCCAC | TGGTTCTTCCGTTGAGGGAC |
| Mouse CXCL10 | TTCCCACCCTACGTCCTT | CTGTCTTCCTCCTCATCTGTAATC |
| Mouse CXCL13 | CTCCAGGCCACGGTATTCTG | CCAGGGGGCGTAACTTGAAT |
| Mouse G-CSF | CAGCCCAGATCACCCAGAATC | GCTGCAGGGCCATTAGCTTC |
| Mouse IL-10 | GGCGCTGTCATCGATTTCTC | ATGGCCTTGTAGACACCTTGG |
| Mouse β-actin | GATATCGCTGCGCTGGTCG | CATTCCCACCATCACACCCT |
| Human TNFα | TCTCGCACCCCGAGTGA | GGAGCTGCCCCTCAGCTT |
| Human IL-1β | AGAAGTACCTGAGCTCGCCA | CTGGAAGGAGCACTTCATCTGT |
| Human IL-6 | CCCCCAGGAGAAGATTCCAA | TCAATTCGTTCTGAAGAGGTGAGT |
| Human MCP1 | CAGCCAGATGCAATCAATGCC | TGGAATCCTGAACCCACTTCT |
| Human CCL3 | CATTCCGTCACCTGCTCAGAA | GCAGCAAGTGATGCAGAGAACTG |
| Human β-actin | ACGTTGCTATCCAGGCTGTG | GAGGGCATACCCCTCGTAGA |
